# PReS-FINAL-2356: A comparison of the American college of rheumatology and the systemic lupus international collaborating clinics classification criteria for systemic lupus erythematosus using data from the uk juvenile systemic lupus erythematosus cohort study

**DOI:** 10.1186/1546-0096-11-S2-P346

**Published:** 2013-12-05

**Authors:** O Lloyd, E Heaf, T Morgan, C Roberts, L McCann, MW Beresford, The UK JSLE Study Group

**Affiliations:** 1 Department of Paediatric Rheumatology, NHS, University of Liverpool, Liverpool, UK; 2 Institute of Translational Medicine (Child Health), University of Liverpool, Liverpool, UK

## Introduction

Significant differences in disease onset and course have been described in adults and children with SLE. Making a diagnosis of JSLE is complex. The United Kingdom JSLE Cohort Study[1] collects demographic and prospective clinical data on patients fulfilling the ACR classification criteria for SLE ≤18 years of age and those with probable evolving disease from time of diagnosis onwards. In 2012 the SLICC group proposed a new classification designed to enhance and improve the diagnosis of SLE. The aim of our study is to determine if the SLICC classification facilitates earlier diagnosis of SLE in a large juvenile cohort.

## Objectives

- To define the number of patients in the UK JSLE Cohort Study not meeting the ACR criteria but identified as having lupus at diagnosis by SLICC classification criteria using data collected at baseline.

- To identify the number of patients who met the ACR criteria at diagnosis but do not meet the SLICC criteria.

## Methods

We compared data collected at diagnosis in two groups of patients, those with ≥4 ACR criteria and those with ≤3 ACR criteria (probable evolving disease, as defined by consultant paediatric rheumatologist). Some SLICC criteria variables could not be applied (eg. toxic epidermal necrolysis, some forms of chronic cutaneous lupus and CH50) because they are not collected in the UK JSLE Cohort dataset. Data were available for 245 patients.

## Results

In our cohort 192 (78.4%) patients met ≥4 ACR criteria and 53(21.6%) had ≤3 ACR criteria at diagnosis. Of those with ≤3 ACR criteria, 31(58.5%) were identified as having lupus by application of the new SLICC criteria. In patients identified at diagnosis by SLICC, one patient met the criteria due to biopsy confirmed lupus nephritis and positive ANA. Of the 192 patients with ≥4 ACR, only three (1.6%) failed to meet the SLICC criteria at diagnosis.

## Conclusion

In our cohort of JSLE patients, application of the SLICC classification criteria increased the chance of a definite diagnosis of JSLE compared to use of ACR criteria. This suggests that use of SLICC criteria, which includes greater weighting of immunological criteria, may improve sensitivity for diagnosis of SLE in children and young people. This is of particular relevance for a paediatric population susceptible to a more aggressive disease process, where an earlier diagnosis could ultimately improve disease outcome. Further studies are needed to assess sensitivity and specificity of SLICC criteria in paediatric lupus.

## Disclosure of interest

None declared.
